# Atypical Infective Endocarditis Presenting With Euthermia and Right Lower Quadrant Abdominal Pain

**DOI:** 10.7759/cureus.68525

**Published:** 2024-09-03

**Authors:** Matthew Carvey

**Affiliations:** 1 Emergency Medicine, Cleveland Clinic, Cleveland, USA; 2 Emergency Medicine, MetroHealth Medical Center, Cleveland, USA

**Keywords:** point-of-care echocardiogram, right renal embolism, systemic inflammatory response syndrome, intravenous drug use, right lower quadrant abdominal pain, infective endocarditis

## Abstract

Infectious endocarditis (IE) is an infection of the heart’s endothelial lining, often stemming from an underlying bacteremia. High-risk populations include intravenous substance users, individuals with structural heart disease, those with intravascular devices, and those with prosthetic heart valves. In the emergency department, IE is often suspected in patients with a fever, known risk factors, and unexplained systemic symptoms due to systemic thromboemboli. We present a case of atypical IE occurring in an afebrile 38-year-old woman with a remote history of intravenous drug use. The patient’s clinical presentation was characterized by systemic inflammatory response syndrome, stabbing-like right lower quadrant abdominal pain radiating to the right lower back and the rest of the abdomen, malaise, fatigue, and an absence of a fever. A CT scan revealed a right renal embolism and an infarcted right kidney, prompting a bedside point-of-care echocardiogram that showed a large vegetation on the mitral valve, suggestive of IE with systemic thromboembolic disease. The patient received broad-spectrum antibiotics and antipyretics and ultimately underwent mitral valve replacement, with good recovery upon discharge. Patients with IE are at high risk for life-threatening complications due to tissue damage from systemic microemboli and sepsis. It is important to identify IE’s atypical presentation and risk factors for early recognition, prompt point-of-care echocardiogram, and initiation of treatment. This is particularly important in the era of increased opioid use among our patient population which could potentially conceal an underlying fever.

## Introduction

Infective endocarditis (IE) is a rapidly progressive bacteremia-mediated infection of the endocardium, often linked to intravenous substance use, prosthetic heart valves, rheumatic heart disease, or cardiac defects. Diagnosis traditionally relies on the modified Duke criteria [1], which may be challenging in the emergency department (ED). Diagnosis in the ED often hinges on high-risk historical features and bedside point-of-care echocardiogram findings, especially in acutely septic patients with or without fever. Atypical presentations can complicate diagnosis due to systemic microemboli migration, leading to a myriad of symptoms. Complications of IE include septic shock (10%), systemic thromboembolism (8.4%), acute kidney injury (6.5%), cardiogenic shock (1.5%), and disseminated intravascular coagulation (1.1%) [2]. Prompt recognition of high-risk features by emergency physicians is crucial for timely treatment initiation. Most patients require admission for further evaluation, intravenous antibiotics, and potential valve replacement due to disease severity. We report a case of atypical native mitral-valve IE initially presenting with systemic inflammatory response syndrome-positive criteria, right lower quadrant (RLQ) abdominal pain, malaise, fatigue, and no reported fever, discussing differential diagnoses, triage, and management strategies.

## Case presentation

A 38-year-old woman presented to the ED with stabbing-like RLQ abdominal pain radiating to her right lower back and diffusely across her abdomen, shortness of breath, general malaise, and fatigue. The patient had visited an outside hospital three days prior for acute-on-chronic back pain, received symptomatic management, and was discharged after a negative CT scan of the thoracic and lumbar spine. A review of systems was notable for a mild headache, fatigue, and dyspnea at rest. The patient denied a fever, recent sexual intercourse, dizziness, chest pain, nausea or vomiting, vaginal bleeding or discharge, rectal bleeding, diarrhea, or burning urination. Her past medical history was notable for intravenous substance use, though they stated they had not used any substances in several years. The patient also denied other substance or alcohol use.

The patient presented with tachycardia and acute distress related to her abdominal pain. Initial vital signs were as follows: temperature 36.9°C, blood pressure 130/77 mm Hg, heart rate 100 beats/minute, respiratory rate 24 breaths/minute, and oxygen saturation 99% on room air. The physical examination was notable for significant RLQ abdominal pain with guarding, radiating diffusely to the remainder of the abdomen and right lumbar region. The patient also exhibited dyspnea at rest without adventitious lung sounds. Their neurological examination was unremarkable, with intact strength and sensation in the upper and lower extremities.

**Figure 1:**
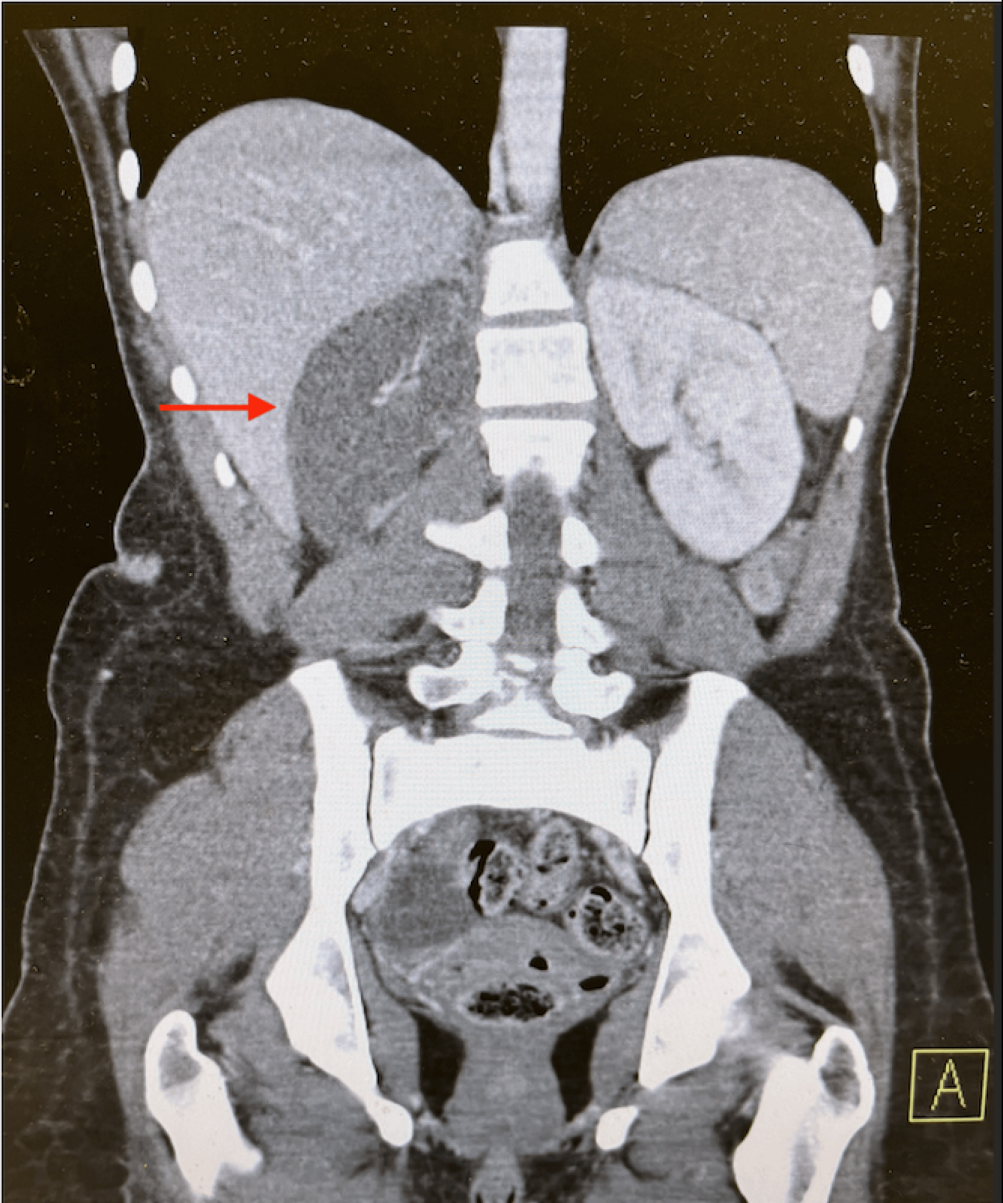
CT scan of the abdomen and pelvis with contrast showing systemic thromboembolic disease with near-global hypoperfusion of the right kidney.

**Figure 2:**
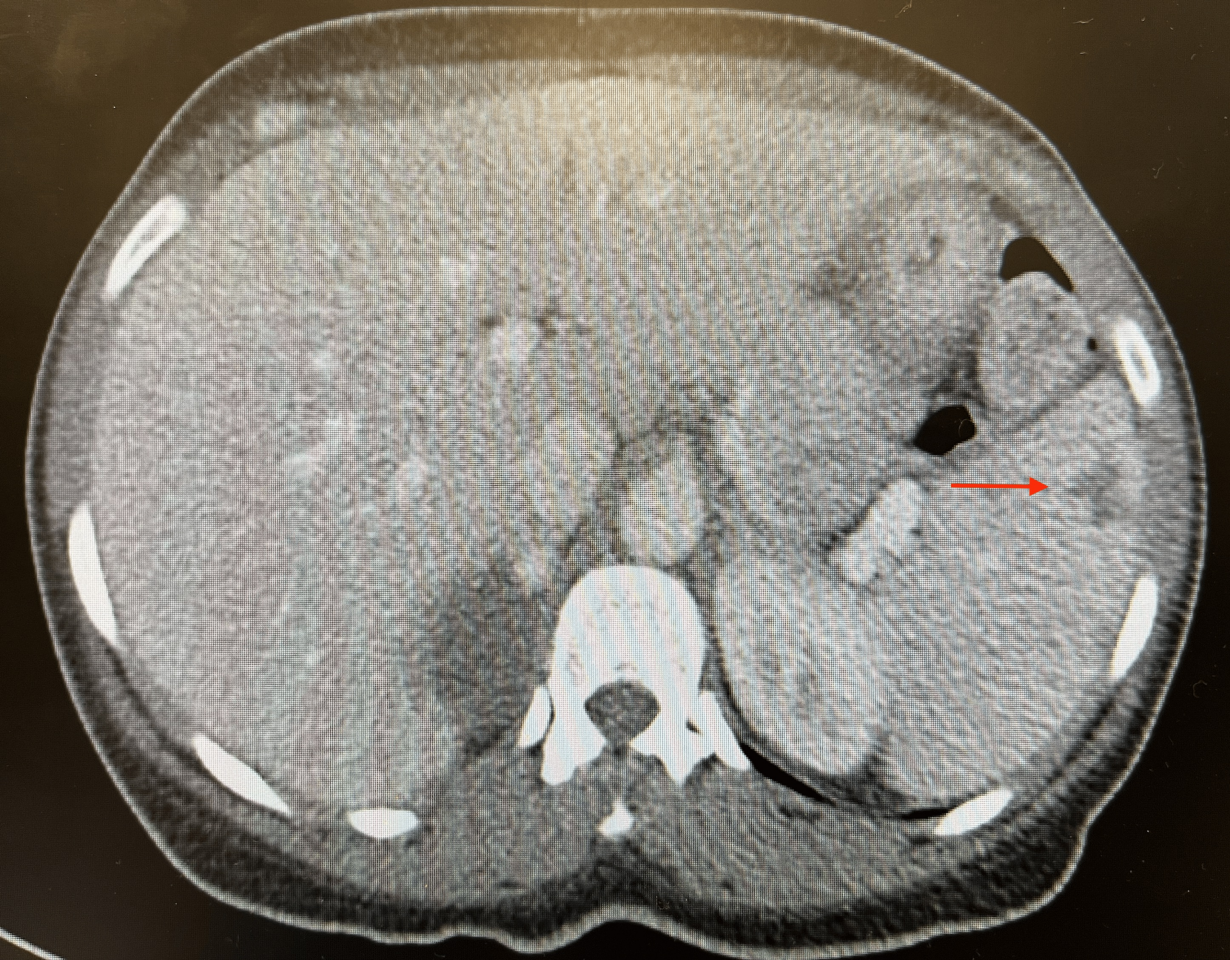
CT scan of the abdomen and pelvis with contrast showing systemic thromboembolic disease with a small multifocal infarct in the spleen.

**Figure 3:**
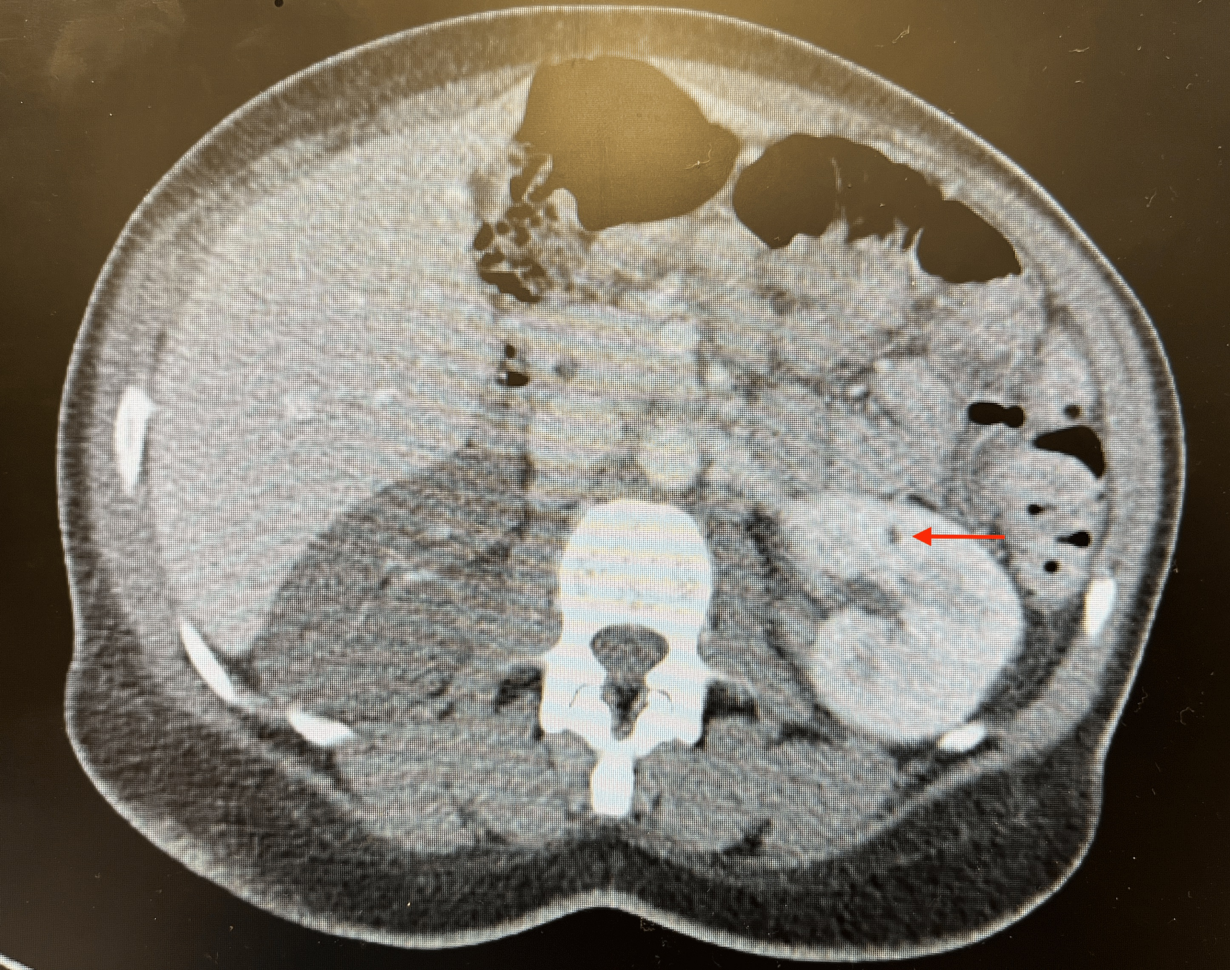
CT scan of the abdomen and pelvis with contrast showing systemic thromboembolic disease with a small multifocal infarct in the left kidney.

**Figure 4:**
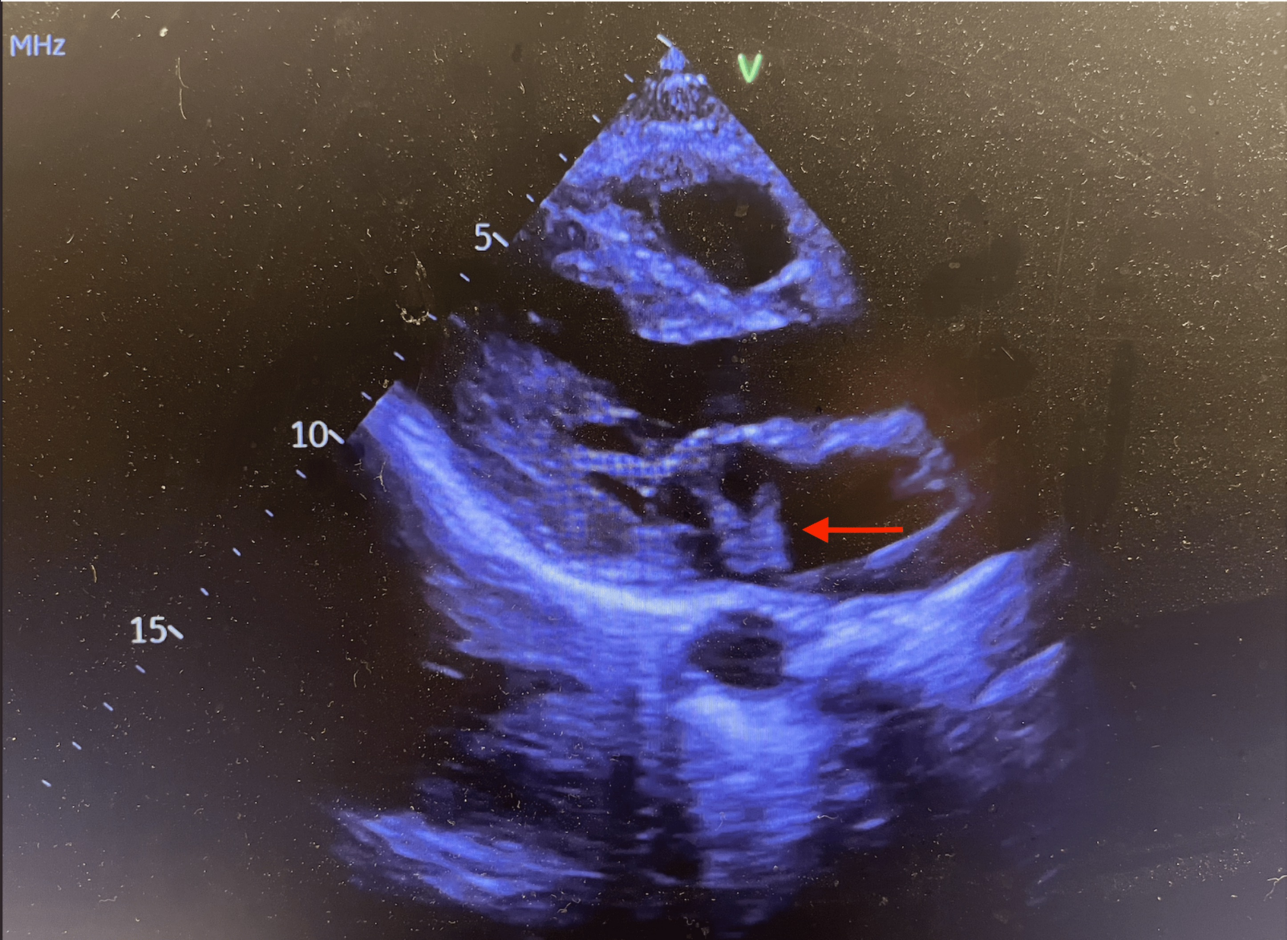
Bedside point-of-care echocardiogram showing a 4.6 cm × 1.3 cm echodensity attached to the anterior leaflet of the mitral valve, traveling freely into the left ventricle and atria, concerning for endocarditis. Significant mitral valve regurgitation is also noted, likely from the anterior leaflet vegetation.

**Figure 5:**
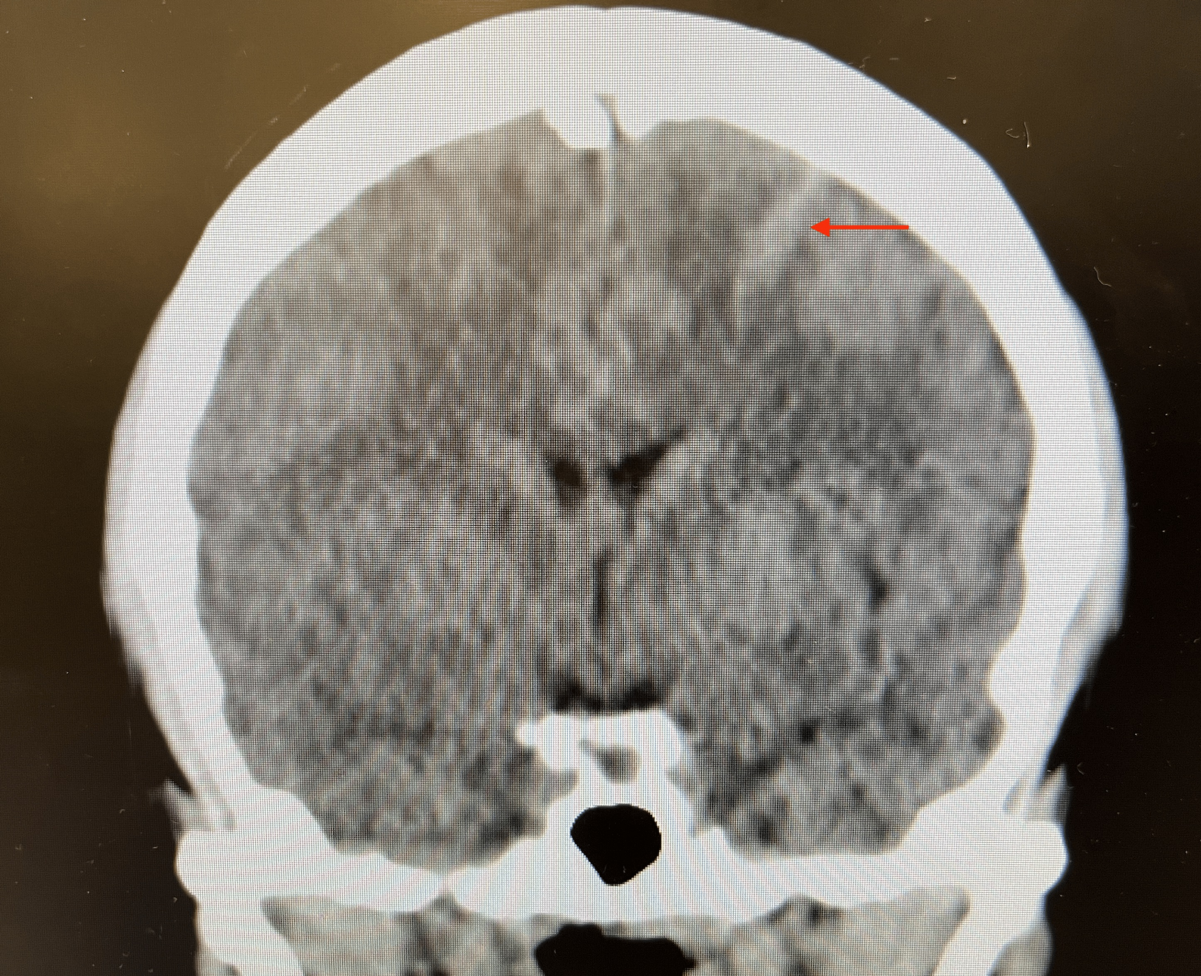
CT scan of the brain without contrast showing a left-sided subarachnoid hemorrhage suspicious in this setting for a possible mycotic aneurysm.

Although the patient presented with a vague history and physical examination, further investigation into the cause of her presentation was necessary. Initial laboratory results are presented in Table 1. Urinalysis was also noted to be positive for nitrites, leukocyte esterase, and white blood cells. Urine toxicology was positive for cocaine and opiates. A CT scan of the abdomen and pelvis with contrast showed systemic thromboembolic disease with near-global hypoperfusion of the right kidney (Figure 1), small multifocal infarcts in the spleen (Figure 2) and left kidney (Figure 3), and probable right renal embolism in the mid-right renal artery. On cardiac auscultation, a holosystolic murmur at the apex was heard. This prompted a bedside point-of-care echocardiogram, which showed a mobile echodensity attached to the anterior leaflet of the mitral valve, traveling freely into the left ventricle and atria, concerning for endocarditis (Figure 4). A CT scan of the brain showed a small acute left frontal sulcal subarachnoid hemorrhage anterior to the central sulcus, concerning for a mycotic aneurysm with hemorrhagic transformation (Figure 5).

**Table 1:** Laboratory results on initial presentation.

Laboratory value	Results (SI)	Reference range (SI)
White blood cell	31.11 × 10^9^/L	4.5–11 × 10^9^/L
Neutrophil	90.40%	50–70%
Lymphocyte	3.40%	20–40%
Monocyte	3.60%	2.0–11%
Eosinophil	0.20%	1.0–4.0%
Basophil	0.50%	0.5–1.0%
Hemoglobin	8.01 mmol/L	7.4–9.9 mmol/L
Platelet count	26 × 10^9^/L	150–450 × 10^9^/L
Sodium	130 mEq/L	135–145 mEq/L
Potassium	3.2 mEq/L	3.4–5.0 mEq/L
Creatinine	97.2 µmol/L	44–97 µmol/L
Blood glucose	4.98 mmol/L	3.9–6.1 mmol/L
Lactate	1.4 mmol/L	0.5–1.6 mmol/L
Bilirubin	398 µmol/L	5.1–17 µmol/L
Alkaline phosphatase	254 U/L	33–136 U/L
Aspartate aminotransferase	93 U/L	9–39 U/L
Alanine aminotransferase	61 U/L	10–52 U/L

The patient was admitted and received acetaminophen 650 mg, vancomycin 1 g, Zosyn 3.375 g, and intravenous fluids while in the ED. On hospital day one, cardiothoracic surgery was consulted, and the patient underwent emergent bioprosthetic mitral valve replacement for endocarditis. Neurosurgery was consulted inpatient and ultimately recommended against surgical intervention. Instead, they advised continuing medical management with continuous neurological examinations for a likely mycotic aneurysm. A total of two blood cultures were collected and returned positive for group B *Streptococcus* without resistance to antibiotics on days one and two of inpatient admission, and returned negative on day five. After consultation with infectious disease on day three, they recommended de-escalation of the antibiotic regimen to ceftriaxone 1 g every 12 hours for a total of four weeks. The patient’s symptoms improved over two weeks, and they were discharged to a skilled nursing facility shortly afterward on continued parenteral antibiotic therapy.

## Discussion

We describe the case of a 38-year-old woman who developed stabbing-like RLQ abdominal pain radiating to their right lower back and diffusely across their abdomen, worsened with palpation, along with shortness of breath, general malaise, and fatigue in the setting of IE. This is thought to have been an atypical variant of IE, characterized by the absence of classical fever but with suspicion for septic emboli. According to Murdoch et al., a fever over 38.0°C is present as an associated symptom in up to 95% of all patients diagnosed with IE, and recreational intravenous substance use represents approximately 10% of cases [3]. Our patient presented without a fever, with vague symptoms of RLQ abdominal pain, malaise, and fatigue, and initially denied the recent use of intravenous substances. The absence of a fever may have been due to the current use of opioids, as indicated by a positive toxicology screen. As stated by Graczyk et al., larger doses of opioids can cause hypothermia by stimulating kappa-type receptors [4], potentially leading to this patient’s afebrile presentation. Other causes of an afebrile presentation in IE include intermittent fever, culture-negative endocarditis, antipyretic use, an immunocompromised state, or advanced age. What makes this patient’s afebrile state unique is that their blood culture returned positive for group B *Streptococcus* with evidence of systemic involvement, they were not on an antipyretic before administration in the ED and were middle-aged. However, the patient also met other criteria for systemic inflammatory response, which necessitated the use of antibiotics despite the lack of a fever.

The presence of RLQ abdominal pain in a woman has many differential diagnoses, including appendicitis, ovarian torsion, ruptured ectopic pregnancy, ureterolithiasis, pyelonephritis, inflammatory bowel disease, or malignancy [5]. Vascular causes of abdominal pain, such as acute mesenteric ischemia, can also present with severe and sudden-onset abdominal pain, although this may lead to an unrevealing abdominal examination [6]. Obstetric processes involving the ovary or fallopian tube were unlikely, as the patient denied recent sexual intercourse, vaginal bleeding, or prior history of ovarian cysts. Renal sources of vague abdominal pain can also present with severe and sudden-onset RLQ pain; however, there were no complaints of dysuria, radiation of pain to the groin, hematuria, or urinary frequency [7].

In most of the diagnoses listed above, a CT of the abdomen and pelvis can confirm the diagnosis. In our case, systemic thromboembolic disease, right kidney hypoperfusion, and small multifocal infarcts in the spleen and left kidney were observed. According to Erdem et al., 44.4% of patients with IE have major embolic events, with 2.9% involving the renal system [8]. This was the primary indication to obtain a bedside point-of-care echocardiogram, which has a specificity of over 90% in diagnosing valvular endocarditis [9,10]. This study showed a 4.6 cm × 1.3 cm mitral valve vegetation without tricuspid valve lesions. Given the strong suspicion for septic thromboemboli on the CT scan and confirmation of IE by bedside point-of-care echocardiogram, further diagnostic evaluation was not needed in the ED.

Complications of IE are secondary to the underlying valvular vegetation and include congestive heart failure, periannular abscesses, systemic embolization, and mycotic aneurysms [11]. Therefore, rapid recognition of an underlying valvular lesion with point-of-care echocardiography in the ED is crucial to initiate antibiotic therapy and emergent valvular surgery if indicated. Focussing on a detailed history of intravenous substance use, valvular disease, implanted cardiac devices, prosthetic heart valves, and immunocompromised states [12] may assist in urgent diagnosis in conjunction with physical examination findings. Although fever may be absent on the initial workup, antibiotics must be initiated immediately if IE is suspected. Additional complications of IE are related to the area of bacterial seeding, which can involve the neurologic, renal, and musculoskeletal systems [13].

Initial management of left-sided IE involves broad-spectrum antimicrobial therapy covering methicillin-resistant *Staphylococcus aureus* and *Pseudomonas* species, particularly in patients with suspected intravenous substance use [14]. A targeted antimicrobial strategy should be employed once the identified organism is obtained from a blood culture, followed by surgical intervention if indicated, especially in the setting of heart failure, uncontrolled infection, or prevention of embolic events [15]. Treatment should continue in the inpatient setting, and those diagnosed with left-sided IE typically require intravenous antibiotics for up to six weeks [16]. The in-hospital mortality rate hovers around 18%, with one-year mortality reaching up to 40% [17].

The annual incidence of IE is 10/100,000 of the general population [12], with 40% of cases affecting the mitral valve [18,19]. In conjunction with antimicrobial therapy, replacement of the infected mitral valve with a prosthesis may be indicated in patients with severe heart failure, invasion beyond the valve leaflets, recurrent systemic embolization, large mobile vegetations, or persistent sepsis despite adequate antibiotic therapy for more than five to seven days [20]. Prosthetic valve endocarditis is a complication of valve replacement, accounting for 10-30% of all cases, with an incidence of 0.3-1.2% per patient per year [21]. Therefore, a discussion regarding the risks of native valve replacement versus solely medical therapy should be considered.

In this case report, the patient endorsed a remote history of intravenous substance use without prior cardiovascular surgeries. Given the poor clinical history and vague symptomatic presentation, the initial suspicion for IE remained low. A broad differential diagnosis should still be considered for RLQ abdominal pain, and a thorough review of prior risk factors should be conducted. Despite testing positive for opioids on urine toxicology, we cannot determine whether the patient used this illicit drug via an intravenous route, based on their denial during questioning in the ED.

## Conclusions

We present a case of IE, a debilitating disease that requires prompt diagnosis and management. It often presents in febrile septic patients with symptoms related to the migration of septic microemboli. This patient’s presentation was unique in that they were afebrile despite significant findings of culture-positive group B *Streptococcus* with evidence of systemic involvement. Additionally, there was no current use of antipyretics before administration in the ED, and the patient initially denied current opioid use. It is important to recognize historical features that may increase a patient’s risk of contracting IE, such as a history of remote intravenous substance use. Given the rise in intravenous opioid use nationally, physicians should keep IE in mind when evaluating patients with known risk factors who are afebrile on presentation, systemic inflammatory response syndrome criteria, and vague symptoms that may be related to septic microemboli migration.
